# Biological activity and magnetic resonance imaging of superparamagnetic iron oxide nanoparticles-labeled adipose-derived stem cells

**DOI:** 10.1186/scrt191

**Published:** 2013-04-23

**Authors:** Jingjing Fan, Yanbin Tan, Liyong Jie, Xinying Wu, Risheng Yu, Minming Zhang

**Affiliations:** 1 Department of Radiology, 2nd Affiliated Hospital, Medical College, Zhejiang University, 88 Jiefang Road, Hangzhou 310009, China

**Keywords:** Adipose-derived stem cells, Bone marrow mesenchymal stem cells, Superparamagnetic iron oxide nanoparticles, Magnetic resonance imaging

## Abstract

**Introduction:**

No comparative study of adipose-derived stem cells (ADSCs) and bone marrow mesenchymal stem cells (BMSCs) by using superparamagnetic iron oxide nanoparticles (SPIOs)-labeling and magnetic resonance imaging (MRI) has been performed.

**Methods:**

We studied the biological activity and MRI of ADSCs by labeling them with SPIOs and comparing them with BMSCs. After incubating the cells in culture medium with different levels of SPIOs (control group: 0 μg/ml; Groups 1 to 3: 25, 50, and 100 μg/ml) for 24 hours, we compared ADSCs with BMSCs in terms of intracellular iron content, labeling efficiency, and cell viability. Stem cells in the culture medium containing 50 μg/ml SPIOs were induced into osteoblasts and fat cells. Adipogenic and osteogenic differentiation potentials were compared. R_2_* values of MRI *in vitro* were compared.

**Results:**

The results showed that labeling efficiency was highest in Group 2. Intracellular iron content and R_2_* values increased with increasing concentrations of SPIOs, whereas cell viability decreased with increasing concentrations of SPIOs, and adipogenic and osteogenic differentiation potentials decreased. However, we found no significant difference between the two kinds of cells for any of these indexes.

**Conclusions:**

ADSCs can be labeled and traced as easily as BMSCs *in vitro*. Given their abundance and higher proliferative capacity, as was previously shown, ADSCs may be better suited to stem cell therapy than are BMSCs.

## Introduction

Mesenchymal stem cells (MSCs) appear to be ideally suited for therapeutic use in tissue repair and many other diseases that are able to differentiate into various types of tissue cells derived from other embryonic layers [1-3]. Bone marrow mesenchymal stem cells (BMSCs) were the first MSCs used for laboratory research [4]. However, conventional bone marrow procurement procedures are distressing for the patient and yield a low number of MSCs. Bone marrow fat increases with age, which often means a sufficient number of MSCs cannot be extracted. Many studies have thus investigated alternative sources to bone marrow for MSCs.

Adipose tissue, like bone marrow, is derived from the embryonic mesenchyme and represents a rich source of MSCs [5]. Many studies have compared various aspects of cell biology between the two kinds of stem cells. Taléns-Visconti *et al.*[4] showed that adipose-derived stem cells (ADSCs) have a similar hepatogenic differentiation potential to that of BMSCs, but a longer culture period and higher proliferative capacity. Nakao *et al.*[3] found that ADSCs facilitate the homing of mouse stem cells to bone marrow better than do BMSCs. Kern *et al.*[6] found that the proliferative capacity of ADSCs was the highest of all the MSCs. Some researchers have found that ADSCs have lower immunogenicity and immunosuppressive effects, implying a lower rejection and higher success rate in transplantation [7,8]. Furthermore, the number of ADSCs is large and is not affected by age [9], but all the comparisons are in biology aspects. It is not enough for cell choice just to compare the characteristics of these two kinds of stem cells.

Central to the success of stem cell therapy is the ability of cells to migrate and engraft. Magnetic resonance imaging (MRI) is useful for evaluating the ability of cells to migrate and engraft [10-16]. SPIOs have been used to label cells, allowing researchers to monitor cell migration by using MRI in experiments [17-21]. BMSCs were the first kind of MSCs to be labeled with superparamagnetic iron oxide particles (SPIOs) and successfully traced *in vivo*[10,11,19]. Many studies have also used SPIO labeling and MRI to study ADSCs [22-24]. However, a comparative study of ADSCs and BMSCs by using SPIO labeling and MRI has not been performed.

This study investigated whether ADSCs can be labeled and traced as easily as BMSCs, examining the intracellular iron content, labeling efficiency, cell viability, adipogenic and osteogenic differentiation potentials, and MRI of SPIO-labeled ADSCs and compared these with BMSCs, to help guide the selection of cells for stem cell therapy.

## Methods

### Preparation of SPIOs

SPIOs were prepared according to the method developed by Racuciu *et al.*[25] with some modifications. In brief, an aqueous solution of 4.16 g FeCl_2_.4H_2_O and 10.44 g FeCl_3_.6H_2_O in 380 ml demineralized water was mixed under vigorous and continuous stirring with 40 ml 25% NH_4_OH as the precipitant. The precipitated black powder was vibrated ultrasonically for 30 minutes. Five grams of citric acid in 10 ml water was then added to the powder, and the temperature was increased to 90°C with stirring for 60 minutes. The resulting black powder was washed several times until neutral and removed by decanting. The iron content was measured by using a total iron reagent set (Pointe Scientific, Canton, MI, USA). The shape and bare core size of SPIOs were measured with transmission electron microscopy (TEM) (JEM-1230; JEOL, Japan). The diameter and polydispersity index (PDI) of SPIOs were measured with Zetasizer Nano (S90, Malvern, UK).

### Preparation of adipose-derived stem cells

ADSCs were prepared according to the method developed by Zuk *et al.*[5] with some modifications. In brief, subcutaneous adipose tissue (3 to 4 g) was obtained from the inguinal regions of male Sprague–Dawley (SD, Vital River, Beijing, China) rats. The adipose tissue was minced and digested with collagenase I (2 mg/ml; Worthington Biochemical Corp, Lakewood, NJ, USA) at 37°C for 30 to 60 minutes. The digested adipose tissue was filtered twice with a 100-μm and then a 25-μm nylon membrane to eliminate the undigested fragments. The cellular suspension was centrifuged at 1,000 *g* for 10 minutes. The cell pellets were resuspended in cell-culture medium (CCM, DMEM+10%FBS) and cultivated for 24 hours at 37°C in 5% CO_2_. Unattached cells and debris were removed, and fresh CCM containing 15% fetal bovine serum (FBS, Gibco, arlsruhe, Germany) was added to the adherent cells, which were cultured at 37°C in 5% CO_2_. Cell-surface markers were measured with flow cytometry (Beckman FC500, CA, USA). Passage 4 cells were used for the following experiments.

### Preparation of bone marrow stem cells

BMSCs were isolated from bone marrow as described previously [26]. In brief, the bone marrow was harvested from the femurs and tibiae of the same male SD rats as for the ADSCs. Bone marrow cells were resuspended in phosphate-buffered saline (PBS, Gibco) to a final volume of 10 ml and layered over an equal volume of 1.077 g/ml Percoll solution (Pharmacia, Piscataway, NJ, USA). After centrifugation at 2,000 rpm for 20 minutes, the mononuclear cells were recovered and transferred to a 100-mm culture flask (Corning, Schiphol-Rijk, the Netherlands) and incubated (37°C, 5% humidified CO_2_) with low-glucose Dulbecco Modified Eagle Medium (DMEM, Gibco) containing 0.2 mmol/ml L-glutamine (Gibco), 100 U/ml penicillin (Gibco), 100 μg/ml streptomycin (Gibco), 10 ng/ml epidermal growth factor (EGF, PeproTech, Rocky Hill, NJ, USA), and 10% fetal calf serum (PAA, Pasching, Austria). Nonadherent cells were removed after 24 hours. Cell-surface markers were measured with flow cytometry. Passage 3 or 4 cells were used for the following experiments.

#### *Magnetic labeling*

SPIOs were preincubated in CCM and antibiotics for 60 minutes at room temperature. The concentrations of SPIOs in cell-culture medium were 25 μg/ml (Group 1), 50 μg/ml (Group 2), and 100 μg/ml (Group 3), whereas a medium without SPIOs was used for the control group. The two kinds of stem cells were incubated in CCM (37°C, 5% humidified CO_2_) for 24 hours.

#### *Prussian blue staining*

Prussian blue staining was used to detect the presence of iron oxide nanoparticles. Cells of Groups 1 to 3 were fixed in 4% paraformaldehyde for 30 minutes and then detected with Prussian blue staining. In brief, fixed cells were washed 3 times with PBS, incubated for 30 minutes with 2% potassium ferrocyanide in 6% hydrochloric acid, and then rewashed 3 times with PBS. Labeled cells were examined under a light microscope to determine intracellular iron oxide distribution.

#### *Iron content*

Cells labeled as described were washed with culture medium and then washed 3 times with PBS, resuspended in 37% HCl, and incubated at 70°C for 30 minutes. Iron content was determined by using a total iron reagent set. The average iron content per cell was then calculated.

#### *Viability*

To determine cell viability, cells of each group were initially seeded in 96-well plates at 5,000 cells per well. After incubation for 72 hours, cells of each group were assessed by using a standard 3-(4,5)-dimethylthialzo(−z-yl)-2,5-di-phenyltetrazoliumbromide (MTT) assay (Sigma-Aldrich, St. Louis, MO, USA) for 4 hours. The supernatant fluid was discarded, and 150 μl dimethyl sulfoxide (DMSO, Sigma-Aldrich) was added to every well for 10 minutes with shaking. The light absorption of all cells was measured with an enzyme-linked immunosorbent assay (ELISA) reader (BioTek, VT, USA). Results were expressed as relative ratios versus unlabeled cells.

#### *Differentiation*

Adipogenic and osteogenic differentiation were measured to assess the effect of SPIOs on the transdifferentiation potential of cells. Cells in Group 2 and the control group were subjected to two types of induction (adipogenic and osteogenic).

Cells for osteogenic differentiation were seeded in six-well plates at 10^5^ cells per well with CCM. After they reached 60% to 80% confluence, the culture medium was replaced by bone cell-induction culture medium containing 10% FBS, 100 U/ml penicillin/streptomycin, 50 μg/ml L-ascorbate-2-phosphate (Sigma-Aldrich), 0.1 μ*M* dexamethasone, and 10 m*M* β-glycerophosphate in DMEM. The cells were cultured for 2 more weeks. Alizarin red was used to detect matrix mineralization of osteogenic differentiation [18]. Cells were rinsed in PBS, fixed in 4% formaldehyde, and stained in 1% alizarin red solution (Rowley Biochemical Institute, Danvers, MA, USA) for 3 minutes. Stained cells were observed under a phase-contrast microscope (Olympus, Tokyo, Japan). To quantify the change in osteogenic potential, the activity of alkaline phosphatase (ALP) was detected by using an ALP enzyme activity kit [27]. Expression levels of Bone Gla Protein (BGP)-mRNA osteogenic markers were measured with real-time polymerase chain reaction (RT-PCR) [27].

Cells for adipogenic differentiation were seeded in six-well plates at 10^5^ cells per well with CCM. After they reached 60% to 80% confluence, the culture medium was replaced by adipose cell-induction culture medium containing 10% FBS, 100 U/ml penicillin/streptomycin (Sigma-Aldrich), 200 m*M* indomethacin (Sigma-Aldrich), 1 m*M* dexamethasone (Sigma-Aldrich), 0.5 m*M* 3-isobutyl-1-methylxanthine (Sigma-Aldrich), and 10 mg/ml insulin (Sigma-Aldrich) in DMEM. The cells were cultured for 2 more weeks. Cells were rinsed in PBS(PH=7.4), fixed in 4% formaldehyde, and incubated in 2% (wt/vol) Oil Red O (Sigma-Aldrich) for 5 minutes. Stained cells were observed under a phase-contrast microscope (Olympus, Tokyo, Japan). To quantify the change in adipogenic potential, the optical-density (OD) values of lipid droplets stained by Oil Red O were measured. Expression levels of adipocyte Protein 2 (aP2)-mRNA adipogenic markers [28] were measured with RT-PCR.

### Magnetic resonance imaging of SPIO-labeled mesenchyme-derived stem cells *in vitro*

We then determined the differences between the two kinds of SPIO-labeled cells in MR relaxation time. The two kinds of cells labeled with different concentrations of SPIOs were suspended in 1% agarose before being transferred into 1.5-ml microcentrifuge tubes (Eppendorf, Westbury, NY, USA). Each tube contained 1 × 10^4^ cells. The tubes were imaged with an eight-channel phased-array head coil on a 3.0-Tesla MR scanner (GE Signa Excite; GE Medical Systems, WI, USA). Enhanced T_2*_-weighted angiography (ESWAN) sequences were used to enhance the T_2*_ effects of the SPIOs. The sequence parameters were: FOV = 20 × 20 mm^2^; matrix = 240 × 240; TR = 45 msec; NEX = 0.69; slice thickness = 2.0 mm; flip angle = 25 degrees; TE = 5 msec, 9.9 msec, 14.7 msec, 19.6 msec, 24.5 msec, 29.3 msec, 34.2 msec, and 39.0 msec.

### Data analysis

Results were analyzed by using the Student *t* test and one-way ANOVA. *P* < 0.05 was considered statistically significant.

## Results and discussion

### SPIOs and cells

**Figure 1:**
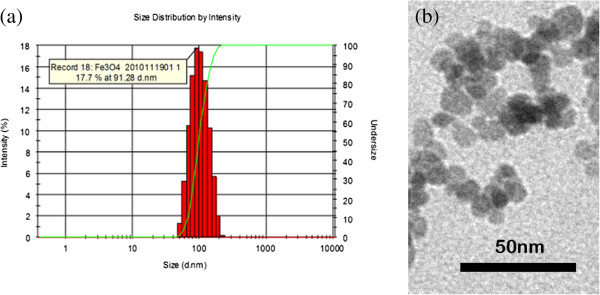
**Identification of SPIOs by Zetasizer Nano and transmission electron microscopy (TEM). (a)** Diameter and PDI of SPIO measured by Zetasizer Nano. **(b)** Shape and bare core size of SPIOs measured with TEM.

**Figure 2:**
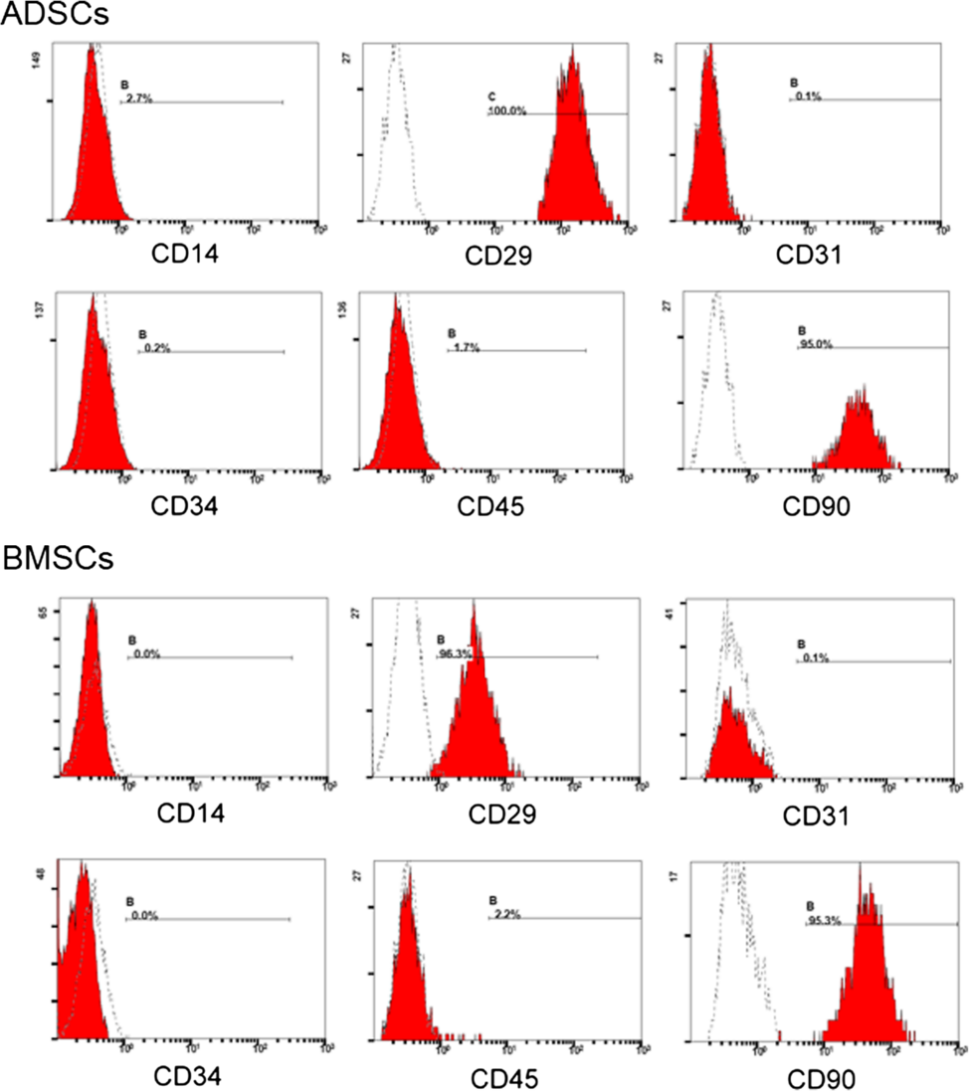
**Identification of adipose-derived stem cells (ADSCs) and bone marrow mesenchymal stem cells (BMSCs) with flow cytometry.** Flow cytometry revealed that the distribution of ADSCs and BMSCs that stained for CD14, CD31, CD34, and CD45 (shaded regions) did not differ from that of the isotype control (open regions). The majority of cells positively stained for CD29 and CD90 (shaded regions) compared with the isotype control cells (open regions).

The citric-acid-coated SPIOs were brownish-black colloid fluids. The iron content was 22.4 mg/ml. The average diameter of SPIOs was 95.58 nm, and the PDI was 0.101, which means the distribution of the SPIO-diameter stenosis was ideal (Figure 1a). The shape of the bare core of SPIOs was round, and the diameter of the bare core was about 8 to 10 nm (Figure 1b). CD14,CD31,CD34,CD45 antigens were found to be negatively expressed in labeled cells, while CD29 and CD90 were found to be positively expressed in labeled cells (Figure 2). These cells thus expressed the same cell surface markers as stem cells, so could be identified as stem cells.

### Labeling efficiency and intracellular iron content

**Figure 3:**
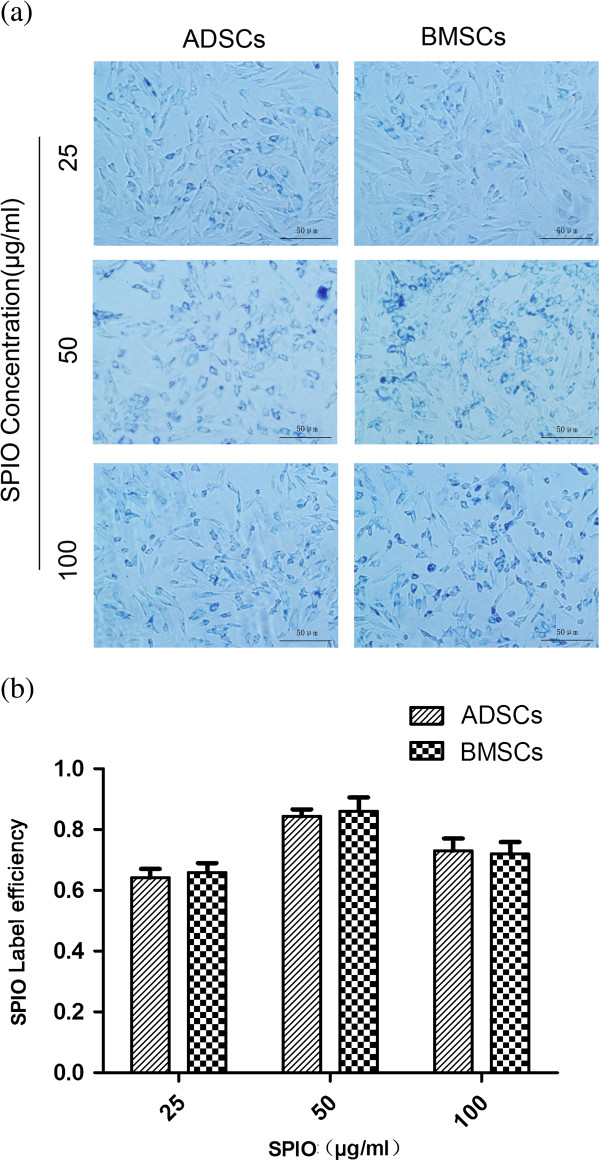
**Measurement iron in cells by Prussian blue staining. (a)** Adipose-derived stem cells (ADSCs) or bone marrow mesenchymal stem cells (BMSCs) were incubated with different concentrations of SPIOs. Then Prussian blue staining was taken to measure the iron content in ADSCs or BMSCs. Blue particles in cytoplasm are SPIOs. **(b)** Labeling efficiency of ADSCs or BMSCs in different concentrations of SPIOs.

After Prussian blue staining, we found blue particles in the cytoplasm of cells. The blue particles were SPIOs surrounding the nucleus (Figure 3a). The labeling efficiency was highest in Group 2 (50 μg/ml), followed by Group 3 and Group 1 (*P* > 0.05). The labeling efficiency of the two kinds of cells showed no significant difference (Group 1: *t* = 1.005, *P* > 0.05; Group 2: *t* = 0.860, *P* > 0.05; Group 3: *t* = 0.492, *P* > 0.05) (Figure 3b).

**Figure 4:**
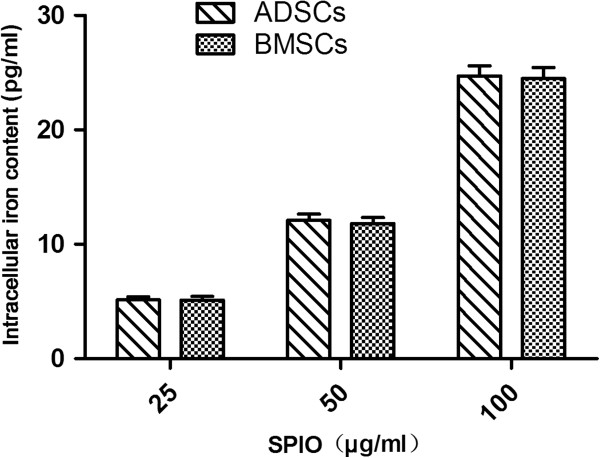
Intracellular iron content measured with a total iron reagent set.

The intracellular iron content increased with increasing concentrations of SPIOs (*P* < 0.05), but no difference was found between the two kinds of cells (Group 1: *t* = 0.020; *P* > 0.05; Group 2: *t* = 0.073; *P* > 0.05; Group 3: *t* = 0.181; *P* > 0.05) (Figure 4). This implies that ADSCs have a similar ability to take up SPIOs to BMSCs. The intracellular iron content of Group 2 ADSCs confirmed that the cells could be clearly traced by MRI *in vivo*, being above the minimum necessary for MRI (5 to 6 pg per cell) [29,30].

### Viability and differentiation

**Figure 5:**
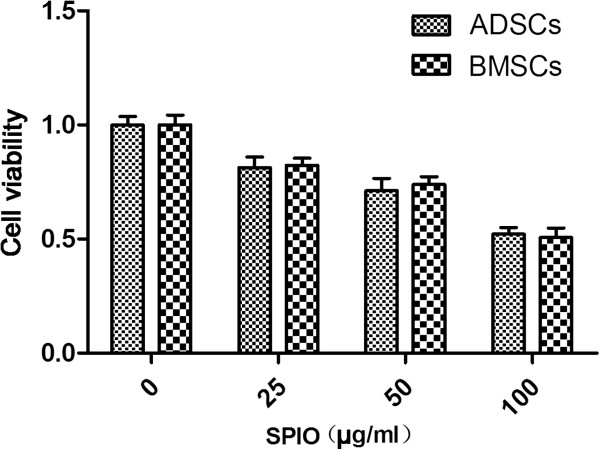
**Viability of cells measured with MTT assay.** Adipose-derived stem cells (ADSCs) or bone marrow mesenchymal stem cells (BMSCs) were incubated with different concentrations of SPIOs, seeded into 96-well microplates, and then the cell viability was measured with the MTT assay.

The viability of cells decreased with increasing concentrations of SPIOs (*P* < 0.05). However, the viability of the two kinds of cells showed no significant difference (Group 1: *t* = 0.533; *P* > 0.05; Group 2: *t* = 1.106; *P* > 0.05; Group 3: *t* = 0.773; *P* > 0.05; control group: *t* = 0.0002; *P* > 0.05) (Figure 5). Because of their higher labeling efficiency and viability, we chose the cells in Group 2 to measure the changes in adipogenic and osteogenic potential.

**Figure 6:**
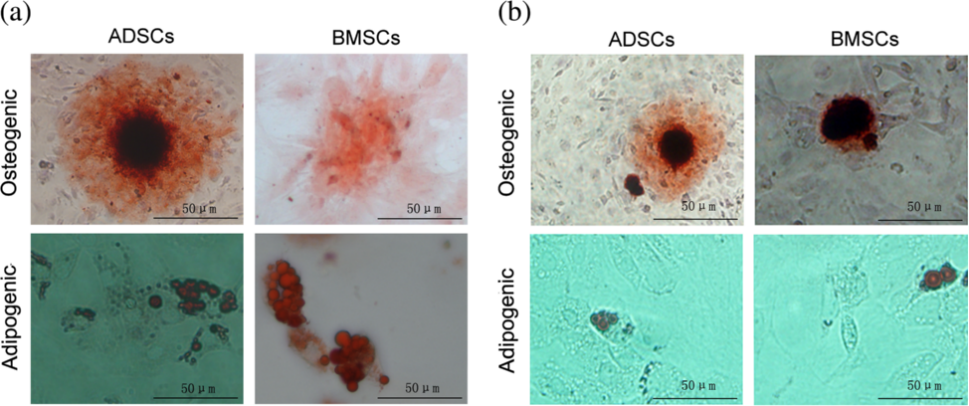
**Differentiation ability of MSCs with or without SPIO incubation. (a)** Adipose-derived stem cells (ADSCs) or bone marrow mesenchymal stem cells (BMSCs) stained in alizarin red solution after osteogenic induction and stained in Oil Red O after adipogenic induction. **(b)** ADSCs or BMSCs were incubated with SPIOs (50 μg/ml), and then the osteogenic ability or adipogenic ability was measured.

**Figure 7:**
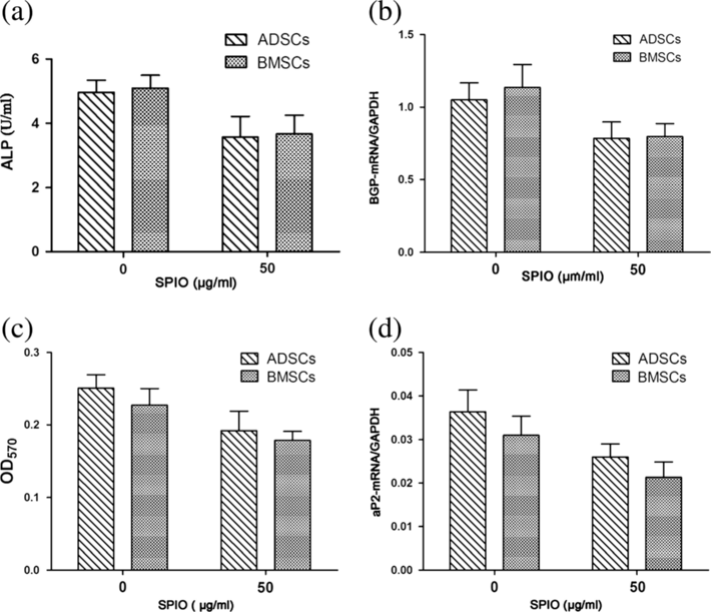
**Analysis of osteogenic and adipogenic ability.** Adipose-derived stem cells (ADSCs) or bone marrow mesenchymal stem cells (BMSCs) were incubated with or without SPIOs (50 ng/ml). **(a)** Activity of ALP in two kinds of cells after osteogenic induction. **(b)** Real-time PCR analysis of BGP-mRNA after osteogenic induction. **(c)** OD value of lipid droplets after adipogenic induction. **(d)** Expression of aP2-mRNA after adipogenic induction.

After induction, phase-contrast microscopy revealed bone nodes and lipid droplets in the cells. Bone nodes became smaller, whereas lipid droplets shrunk in size (Figure 6a and b). The activity of ALP decreased (ADSCs: *t* = 5.433; *P* < 0.05; BMSCs: *t* = 6.217; *P* < 0.05) (Figure 7a), as did the expression of BGP-mRNA (ADSCs: *t* = 4.383; *P* < 0.05; BMSCs: *t* = 5.419; *P* < 0.05) (Figure 7b). Thus, the osteogenic potential of cells after SPIO labeling decreased. The OD value of lipid droplets decreased (ADSCs: *t* = 5.171; *P* < 0.05; BMSCs: *t* = 5.404; *P* < 0.05) (Figure 7c), as did the expression of aP2-mRNA (ADSCs: *t* = 4.992; *P* < 0.05; BMSCs: *t* = 4.830; *P* < 0.05) (Figure 7d). Thus, the adipogenic potential of cells after SPIO labeling decreased. However, the differentiation potential of the two kinds of cells showed no significant difference (*P* > 0.05), (ALP: *t* = 0.210; *P* > 0.05; BGP-mRNA: *t* = 0.156; *P* > 0.05; OD value: *t* = 0.872; *P* > 0.05; aP2-mRNA: *t* = 2.235; *P* > 0.05) (Figure 7a through d). Thus, ADSCs could withstand the toxicity of SPIOs as well as BMSCs.

Many studies have reported that high levels of SPIOs can affect the viability and differentiation of MSCs. Rice *et al.*[24] confirmed that cell viability decreased with increasing concentrations of SPIOs when iron concentrations exceeded 12.5 μg/ml. Arhab *et al.*[31] found that cell viability was affected when iron concentrations of SPIOs reached 50 μg/ml. Wang *et al.*[32] and Kostura *et al.*[33] suggested that SPIOs inhibit chondrogenesis of stem cells at high concentrations, whereas Chen *et al.*[34] assert that SPIOs inhibit osteogenesis of stem cells at high concentrations. Kim *et al.*[22] found that stem cell markers (Oct-4) and cell-surface markers (CD45) changed after SPIO labeling. The mechanism of this effect is unclear. Pawelezyk *et al.*[35] and Karlsson *et al.*[36] suggested the following possible reason: when the intracellular iron concentration is too high, the iron oxide nanoparticles can become toxic to cells through Fenton-type Haber-Weiss reactions caused by free-radical damage [37]. Thus, high levels of SPIOs can affect the viability and differentiation of MSCs. Choosing the ideal concentration of SPIOs and optimizing the physical and chemical characteristics of SPIOs are therefore important in the tracing of transplanted ADCSs. Nevertheless, the viability and differentiation of labeled ADSCs in our experiments appeared to be largely retained, with labeled ADSCs maintaining their “stem cell characteristics,” suggesting that SPIO-labeled ADSCs offer promise for stem cell therapies.

### Magnetic resonance imaging of SPIO-labeled MSCs *in vitro*

**Figure 8:**
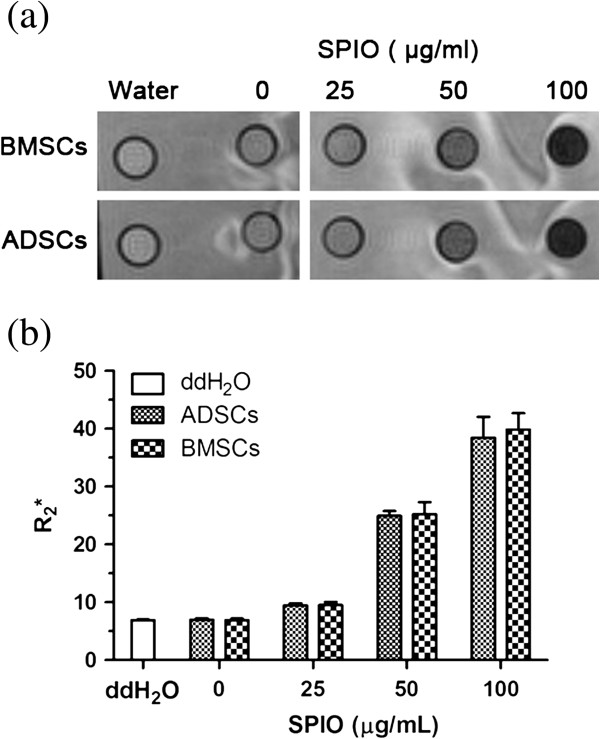
**MRI of SPIO-labeled MSCs *in vitro*. (a)** Adipose-derived stem cells (ADSCs) or bone marrow mesenchymal stem cells (BMSCs) were incubated with different concentrations of SPIO and then scanned with MRI. **(b)** R2* values of ADSCs or BMSCs in different groups were measured.

We found the intensity of the MR signal in control group cells to be similar to that of water. The signal intensity in SPIO-labeled cells decreased with increasing concentrations of SPIOs (Figure 8a). The R_2_* of SPIO-labeled MSCs increased with increasing concentrations of SPIOs (*P* < 0.05), but no significant differences appeared between the two kinds of cells (Group 1: *t* = 0.087; *P* > 0.05; Group 2: *t* = 0.328; *P* > 0.05; Group 3: *t* = 0.798; *P* > 0.05; Group 4: *t* = 0.459; *P* > 0.05) (Figure 8b). Thus, MR imaging of SPIO-labeled ADSCs was virtually identical to that of BMSCs. MRI can reflect changes in intracellular iron concentrations, and so could be used to study changes in SPIO-labeled ADSCs *in vivo*. Further animal experiments should be undertaken to certify the influence of SPIOs on functions of ADSCs and BMSCs *in vivo*.

Our study certified that ADSCs have similar and comparative effects as BMSCs *in vitro*. Moreover, ADSCs have more-abundant sources and greater proliferation ability, compared with BMSCs. Once ADSCs are further confirmed to have effects comparable to those of BMSCs *in vivo*, ADSCs will be a significant option in stem cell application in clinical tracing and treatment.

## Conclusions

In this study, we investigated the biological activity and MRI of SPIO-labeled ADSCs and compared these with BMSCs. We found that SPIO-labeled ADSCs were not statistically significantly different from BMSCs in terms of labeling efficiency, intracellular iron content, cell viability, differentiation, and MR imaging. Thus, ADSCs can be labeled and traced as easily as BMSCs *in vitro*. Given their abundance and higher proliferative capacity, as was previously shown [4-6,9], ADSCs may be better suited to stem cell therapy than BMSCs. When choosing between BMSCs and ADSCs for clinical therapies, these results should be taken into consideration in selecting the best MSCs for the treatment.

## Abbreviations

ADSC: Adipose-derived stem cell; ALP: Alkaline phosphatase; aP2: Adipocyte protein 2; BGP: Bone Gla protein; BMSC: Bone marrow mesenchymal stem cell; FBS: Fetal bovine serum; MRI: Magnetic resonance imaging; PDI: Polydispersity index; SPIO: Superparamagnetic iron oxide nanoparticle.

## Competing interests

The authors declare that they have no competing interests.

## Authors’ contributions

JJF conceived and designed the experiments, performed the experiments, analyzed the data, and wrote the manuscript. YBT performed the experiments and analyzed the data. LYJ conceived and designed the experiments. XYW designed the experiments and analyzed the data. RSY contributed reagents/materials and revised the manuscript. MMZ conceived and designed the experiments, analyzed the data, and revised the manuscript. All authors read and approved the final manuscript.
